# 

**Published:** 1904-02-13

**Authors:** 


THE HOSPITAL. "The standard of highest purity."—*Lancet.* FEBRUARY 13TH, 1904.

**CADBURY'S COCOA**
"A PERFECT FOOD."

**CADBURY'S COCOA**

NO DRUGS OR ALKALIES USED.

### THE HOSPITAL

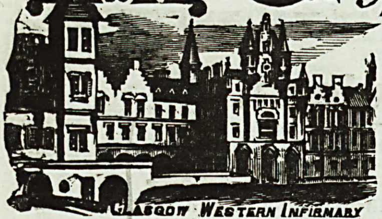

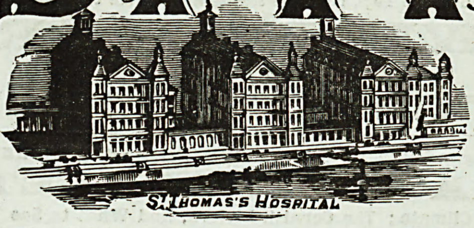

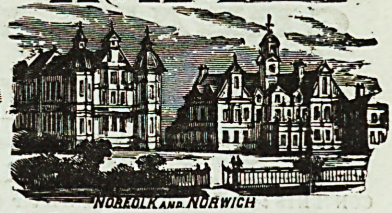

No. 907. Vol. XXXV. [Registered as a Newspaper.] SATURDAY, FEB. 13TH, 1904. [Subscription Terms, see p. 350.] PRICE THREEPENCE.

